# The unique role of *nucS*-mediated noncanonical mismatch repair in *Mycobacterium tuberculosis* resistance evolution

**DOI:** 10.1128/mbio.03310-25

**Published:** 2025-12-22

**Authors:** Isabel Martín-Blecua, Jorge Sastre-Domínguez, José Ramón Valverde, Pablo García-Bravo, Ángel Ruiz-Enamorado, Rafael Prados-Rosales, Lahari Das, William R. Jacobs, Álvaro San Millán, Jesús Blázquez, Sonia Gullón

**Affiliations:** 1 Department of Microbial Biotechnology, Centro Nacional de Biotecnología-CSIC, Madrid, Spain; 2 Scientific Computing Service, Centro Nacional de Biotecnología-CSIC, Madrid, Spain; 3 Department of Preventive Medicine, Public Health, and Microbiology, School of Medicine, Universidad Autónoma de Madrid 16722, Madrid, Spain; 4 Department of Microbiology and Immunology, Albert Einstein College of Medicine 2006, Bronx, New York, USA; Corporación CorpoGen, Bogotá D.C, Colombia

**Keywords:** *Mycobacterium tuberculosis*, NucS/EndoMS, hypermutation, DNA repair, antibiotic resistance, genome stability, non-canonical mismatch repair, mutation rate

## Abstract

DNA surveillance mechanisms are crucial for maintaining genome stability and minimizing mutation rates. Mismatch repair (MMR) corrects replication errors that escape DNA-polymerase proofreading. In most organisms, MMR is orchestrated by MutS and MutL proteins. However, certain Archaea and Actinobacteria, including the major human pathogen *Mycobacterium tuberculosis*, lack these components. Instead, they appear to rely on the nuclease EndoMS/NucS, a structurally distinct enzyme governing a non-canonical MMR pathway. Since *M. tuberculosis* acquires drug resistance exclusively through chromosomal mutations, understanding its mutation rate regulation is critical. Nevertheless, the role of NucS in drug resistance evolution remains largely unexplored. We investigated NucS function in *M. tuberculosis* and uncovered a unique resistance dynamic distinct from other Actinobacteria. Deleting *nucS* altered the mutational spectrum but had minimal impact on the emergence of antibiotic-resistant mutants, contrasting sharply with other Actinobacteria where *nucS* loss dramatically increases mutation rates. This atypical behavior cannot be attributed solely to the R144S NucS polymorphism present in the H37Rv reference strain. Introducing the consensus NucS sequence in H37Rv produced only subtle changes in mutational spectrum without major effects on mutation rates. Analysis of 44,921 *M*. *tuberculosis* genomes revealed that most R144S-containing strains belong to the Euro-American sub-lineage 4.9, with no significant association with antibiotic resistance. However, *nucS* is under strong purifying selection, and R144S changes arose independently during *M. tuberculosis* evolution (homoplasy). Overall, our findings challenge the view that *nucS* serves as a genome stability guardian in *M. tuberculosis* and suggest additional mismatch repair mechanism(s) beyond NucS in this pathogen.

**IMPORTANCE:**

DNA repair systems are crucial for maintaining the integrity of genetic information by scanning and correcting errors that arise during DNA replication. Most organisms use well-characterized proteins (MutS and MutL) for this task, but some bacteria and archaea, including most Actinobacteria, lack these conventional components. Instead, they employ an alternative enzyme, NucS, to fix replicative DNA errors. This alternative pathway is thought to limit harmful mutations and, in turn, reduce the likelihood of drug resistance development. In our study of *Mycobacterium tuberculosis*, the pathogen responsible for tuberculosis, we found that NucS plays only a minor role in preventing resistance-associated mutations. This unexpected result challenges current assumptions about DNA repair in this pathogen and points to the existence of other, yet unidentified mechanisms that safeguard its genome. Understanding these processes could open new avenues for therapeutic strategies against tuberculosis, a disease that remains a major global health threat.

## INTRODUCTION

To maintain genome stability, the cells use a number of DNA surveillance and correction processes. Among these processes, the fidelity of DNA replication is a key factor in keeping mutations at a low rate. This fidelity is ensured by both base selection and the 3′−5′ proofreading activity of replicative DNA polymerase, as well as post-replicative mismatch repair (MMR). In *Escherichia coli,* the contribution of each of these activities to the error rate is roughly estimated at 10^−5^ to 10^−6^, 10^−1^ to 10,^−2^ and 10^−2^ to 10^−3^, respectively, accounting for an overall error rate of about 10^−10^, although these values may vary depending on the genetic marker used (1, 2). MMR repairs errors (mismatches) that escape the correcting activity of DNA polymerase, relying on proteins of the MutS and MutL families for function (3). However, certain archaeal and most actinobacterial species, including the causative agent of tuberculosis (TB), *Mycobacterium tuberculosis*, lack the *mutSL* genes yet exhibit low mutation rates, suggesting the existence of an alternative MMR system (4). The EndoMS/NucS protein (hereafter referred to as NucS for simplicity) appears to play a key role in this alternative MMR pathway in these prokaryotes (4–6). Initially identified in the archaeal species *Pyrococcus abyssi*, NucS is a two-domain protein with an N-terminal DNA-binding domain connected by a short linker to the C-terminal catalytic domain, defining a new family of structure-specific DNA endonucleases (7). Further studies characterized its biochemical activity, revealing that archaeal NucS is an endonuclease that recognizes mismatched sites arising during DNA replication. Once a mismatch is recognized, NucS introduces double-strand breaks (DSBs) flanking the site of the mismatch, generating 5′ sticky ends with five overhanging nucleotides, leaving the mismatched base in the middle. The cleavage activity of the endonuclease NucS was shown to be enhanced by the sliding clamp (PCNA in Archaea and β-clamp in Bacteria) (8, 9). The interaction between NucS and β-clamp occurs through a sequence of five amino acid residues present at the end of the C-terminal NucS sequence. These DSBs must be processed to restore the correct base pair, although the specific repair mechanism remains undefined. Recent studies suggest that neither homologous recombination (HR) nor non-homologous end joining (NHEJ) participates in this pathway in *Mycobacterium smegmatis* (10, 11).

The first *in vivo* evidence of NucS-mediated MMR activity in Actinobacteria was provided by Castañeda-García et al. in 2017 (4), who identified *nucS* (*MSMEG_4923*) as an antimutator gene in *M. smegmatis* through a transposon mutagenesis screen for mutants with high rates of spontaneous rifampicin resistance. The deduced amino acid sequence of this gene had a 27% sequence identity with NucS from *P. abyssi*. Genetic and biological analyses showed that the *nucS*-null (Δ*nucS*) variant of *M. smegmatis* exhibited dramatically increased mutation rates, a transition-biased mutational spectrum, and elevated recombination rates between non-identical (homeologous) DNA sequences, phenotypes almost identical to those produced by the MMR deficiency in other organisms relying on MutS and MutL activity (4). The hypermutator phenotype of actinobacterial *nucS*-null mutants was confirmed in *Streptomyces coelicolor* (4) and further verified in *Corynebacterium glutamicum* (5, 6), *Mycobacterium abscessus* (12), and *Streptomyces ambofaciens* (13). Purified NucS from *C. glutamicum* (5, 6) and *S. ambofaciens* (13) demonstrated enzymatic activity similar to their archaeal counterparts, efficiently correcting transition mutations. NucS was found to efficiently correct mainly transition mutations (A:T>G:C and G:C>A:T) in Actinobacteria *in vivo*, as deduced from analysis of mutations conferring antibiotic resistance (4, 5) and mutation accumulation (MA) experiments (6, 14). Fluctuation tests or estimation of mutant frequency of rifampicin-resistant (RIF-R) spontaneous mutants showed that deletion of *nucS* conferred a hypermutable phenotype across all tested Actinobacteria (4–6, 12, 13), increasing mutation rate by two orders of magnitude compared to wild-type strains (very similar increases to those obtained in other bacteria with a defective MutSL-based MMR like, for instance, *Pseudomonas aeruginosa* for RIF-R mutants (15)). This underscores the critical role of NucS in regulating genome stability and contributing to the evolution and adaptability of these clinically and industrially important species.

*M. tuberculosis* remains one of the deadliest pathogens in human history, causing tuberculosis (TB). TB has recently regained its status as the world’s leading infectious disease killer, with more than 10.8 million cases and 1.25 million deaths in 2023 (16). The increase in antibiotic resistance in *M. tuberculosis* has worsened this public health challenge, complicating treatment protocols and undermining global TB control efforts worldwide. Multidrug-resistant strains (MDR), some of them resistant to most or all effective antibiotics, have emerged steadily over decades (17, 18). Recently, RIF-R *M. tuberculosis* has been included in the 2024 WHO list of bacterial priority pathogens (19). Unlike most bacterial pathogens that acquire drug resistance mainly by horizontal gene transfer, *M. tuberculosis* develops drug resistance exclusively through chromosomal mutations (20), emphasizing the importance of understanding mutation rate regulation in this species (21). There is compelling evidence that hypermutable strains, often associated with defects in MMR components, play a significant role in the development of antibiotic resistance, virulence, persistence, and transmissibility in chronic bacterial pathogens, such as *Pseudomonas aeruginosa* in cystic fibrosis patients (22, 23). Even small increases in mutation rates can have substantial effects on the evolution of antibiotic resistance (21, 24, 25). However, the role of *nucS* in regulating the development of antibiotic resistance in *M. tuberculosis* has not yet been explored. To investigate this, we have constructed and analyzed a Δ*nucS* mutant and its corresponding *nucS*-complemented variant in the *M. tuberculosis* H37Rv model strain. Using fluctuation tests, we assessed the rate at which *M. tuberculosis* acquires resistance to the first-line antitubercular drugs rifampicin (RIF), isoniazid (INH), and ethambutol (EMB). Furthermore, as H37Rv harbors a unique polymorphism (R144S) relative to the *M. tuberculosis* consensus NucS sequence (26), we constructed an engineered variant of H37Rv harboring the NucS consensus sequence and analyzed how it affected the mutational spectrum and resistance rates. Finally, the prevalence of R144S polymorphism among clinical *M. tuberculosis* genomes and its association with drug resistance were analyzed.

## RESULTS

### Deletion of *nucS* does not affect the rate of RIF-R spontaneous mutations

**Fig 1:**
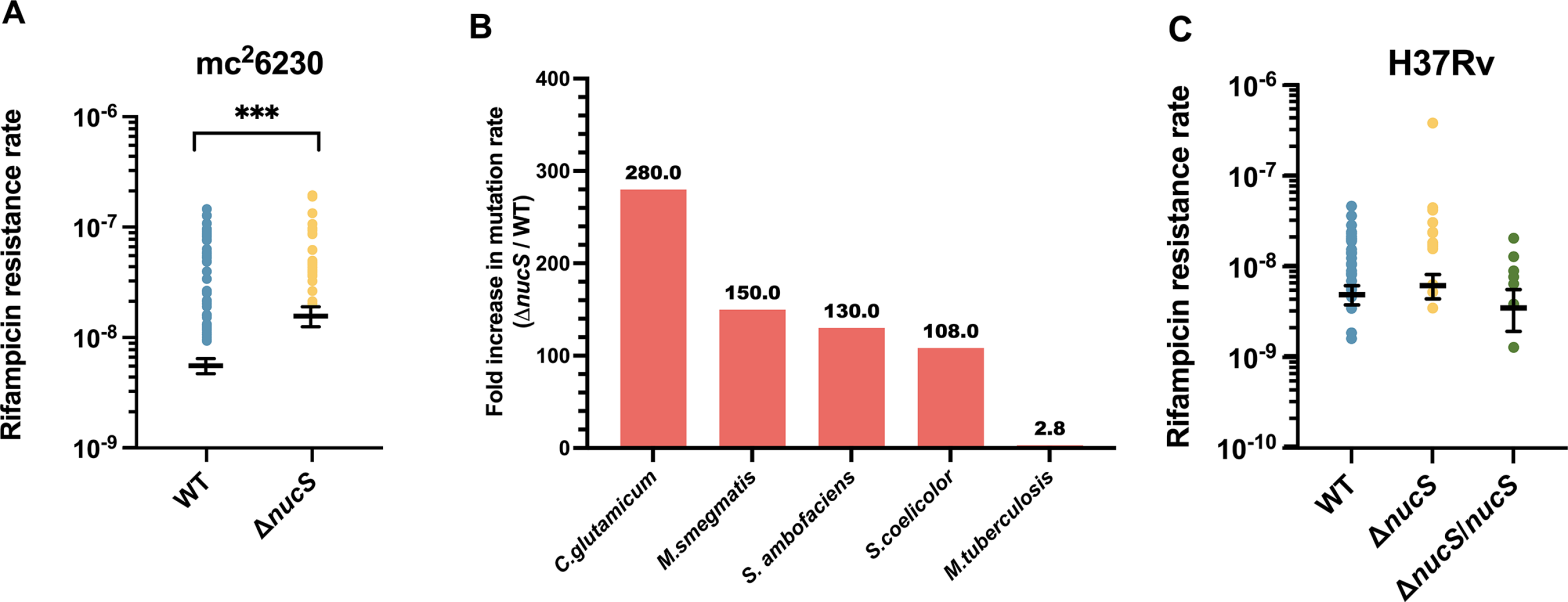
Effect of *nucS* deletion on the rate of spontaneous RIF-R mutants. Mutation rate for RIF-R spontaneous mutants in strain mc^2^6230 and its Δ*nucS* derivative. (**A**) Fold increase for RIF-R in mutation rate for Δ*nucS* derivatives of *M. smegmatis* (4), *S. coelicolor* (4), *S. ambofaciens* (13), *C. glutamicum* (5, 6, 28), and *M. tuberculosis* strain H37Rv mc^2^6230 (this work). ***, *P* < 0.001. (**B**) RIF-R mutation rates of strains H37Rv and their derivatives Δ*nucS* and Δ*nucS*/*nucS* (**C**). Differences are not statistically significant in panel **C**.

To assess the impact of NucS on mutation rates in *M. tuberculosis*, we constructed a Δ*nucS* derivative in the *M. tuberculosis* strain mc^2^6230, an attenuated variant of the model strain H37Rv (27). Deletion of *nucS* produced only a small, although significant, 2.83-fold increase in the rate of RIF-R spontaneous mutations (significance defined by nonoverlapping 99% CI). Specifically, the rates of spontaneous RIF-R mutants were 5.49 × 10^−9^ (95% CI: 4.65–6.37 × 10^−9^) for wild-type (WT) and 1.55 × 10^−8^ (95% CI: 1.24–1.89 × 10^−8^) for the Δ*nucS* mutant (Fig. 1A and Table S1). These results were highly unexpected, given that deletion of *nucS* in all tested Actinobacteria, including *M. smegmatis* (4), *S. coelicolor* (4), *S. ambofaciens* (13), and *C. glutamicum* (5, 6, 28), consistently led to a two-order magnitude increase in spontaneous RIF-R mutation rates (Fig. 1B).

To determine whether this non-mutator phenotype was specific to the H37Rv mc^2^6230 avirulent derivative, we generated a Δ*nucS* mutant in the virulent parental strain H37Rv and analyzed RIF-R mutation rates in wild-type, Δ*nucS* (H37Rv Δ*nucS*), and its *nucS*-complemented derivative (H37Rv Δ*nucS*/*nucS*). Again, the Δ*nucS* strain exhibited a RIF-R mutation rate comparable with the wild-type strain. Specifically, the rates of spontaneous RIF-R mutants were 4.66 × 10^−9^ (95% CI: 3.58–5.86 × 10^−9^), 5.88× 10^−9^ (95% CI: 4.18–7.79 × 10^−9^) and 3.33 × 10^−9^ (95% CI: 1.82–5.29 × 10^−9^) for the wild-type, Δ*nucS,* and Δ*nucS*/*nucS* variants, respectively (Fig. 1C and Table S1). Similar results with the H37Rv Δ*nucS* strain were observed by K. Murphy and C. Sassetti (personal communication). These findings challenged the hypothesis that *nucS* serves as a guardian of genome stability in *M. tuberculosis*.

### Effect of *nucS* deletion on the rate of INH-R and EMB-R spontaneous mutations

**Fig 2:**
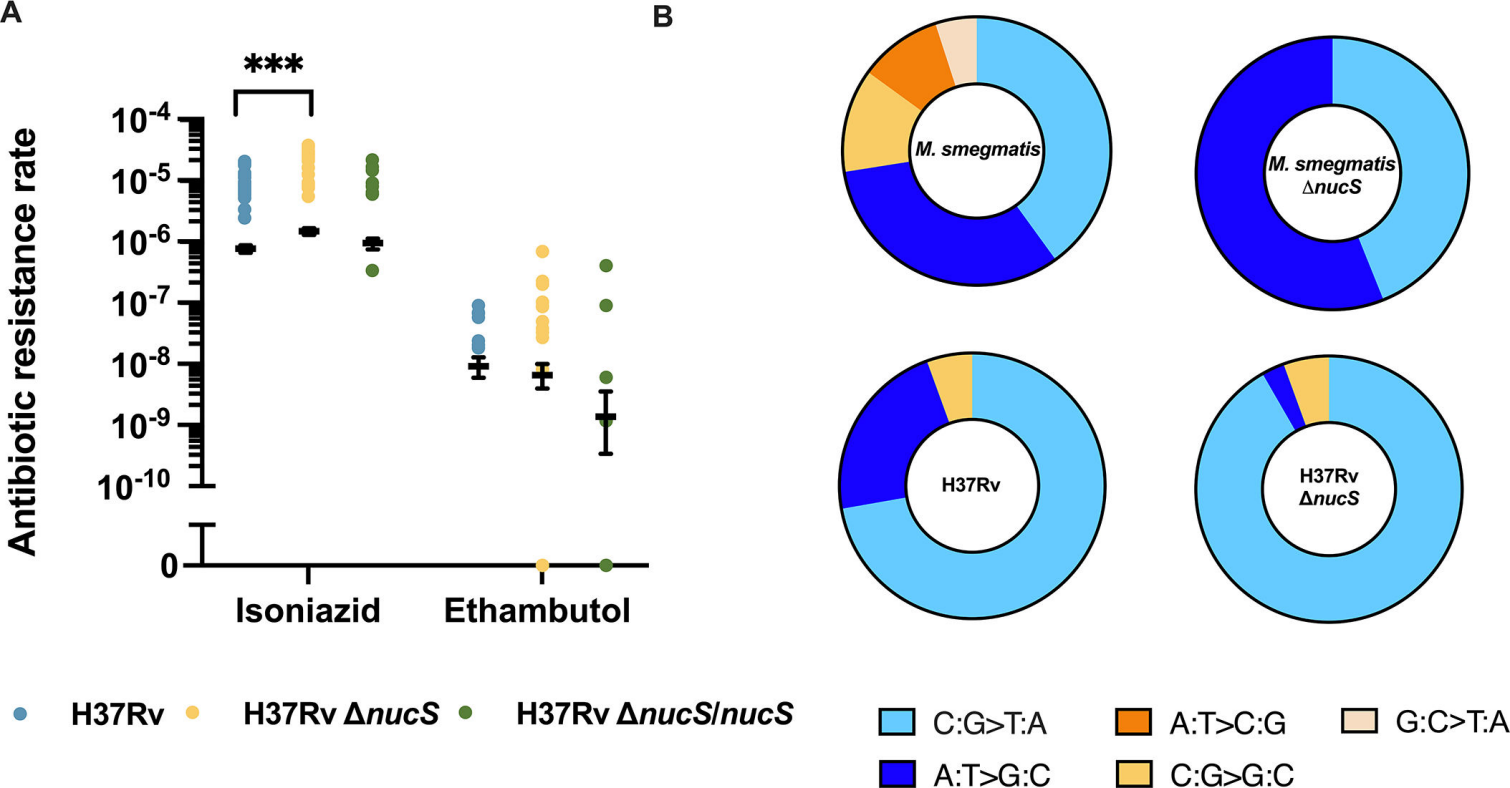
Effect of *nucS* deletion on the rate of spontaneous INH-R and EMB-R mutants in *M. tuberculosis* strains H37Rv and its derivatives (**A**) and mutational spectrum of strains H37Rv and its Δ*nucS* derivative, compared with those of *M. smegmatis* WT and Δ*nucS* (4) (**B**). Asterisks denote statistical significance (defined by nonoverlapping 99% CI): ***, *P* < 0.001. Spectra are expressed as a percentage of RIF-R mutations. Transitions are shown in blue tones, transversions in orange tones. Between 1 and 5 colonies from each fluctuation test culture were pooled. Only colonies with different mutations from the same culture were considered for this study (Table S3).

To further evaluate the role of NucS in modulating mutation rates in *M. tuberculosis*, we tested the mutator phenotype using two additional first-line antitubercular drugs: isoniazid and ethambutol. The spontaneous INH-R mutation rates were 8.09 × 10^−7^ (95% CI: 6.95–9.20 × 10^−7^), 1.57 × 10^−6^ (95% CI: 1.37–1.76 × 10^−6^), and 9.96 × 10^−7^ (95% CI: 7.89–11.9 × 10^−7^) for WT, Δ*nucS,* and *nucS*-complemented H37Rv strains, respectively (Fig. 2A and Table S1). This represented a modest 1.94-fold increase in mutation rate upon *nucS* deletion, with restoration of near WT levels upon complementation. Similarly, the rates of spontaneous EMB-R mutations were 9.57 × 10^−9^ (95% CI: 6.20–13.30 × 10^−9^), 6.88 × 10^−9^ (95% CI: 4.10–10.40 × 10^−9^), and 1.42 × 10^−9^ (95% CI: 0.35–3.67 × 10^−9^) for WT, Δ*nucS,* and *nucS*-complemented H37Rv strains, respectively (Fig. 2A and Table S1). The small differences in mutation rates cannot be attributed to variations in drug susceptibility, as minimum inhibitory concentrations (MICs) were comparable across all strains (Table S2), nor growth rates (Fig. S1), as growth rates were similar for H37Rv and its Δ*nucS* derivative. Taken together, these data indicate that *nucS* does not significantly influence the rate of resistance acquisition in *M. tuberculosis*.

### Loss of *nucS* modifies the spectrum of transitions

To investigate the role of NucS in modulating base pair substitutions (BPSs) spectra in *M. tuberculosis*, we analyzed RIF-R colonies by sequencing the rifampicin resistance-determining region (RRDR) of the *rpoB* gene (Table S3). In the H37Rv WT strain, the BPS spectrum is heavily biased toward transitions, which account for 94.4% (34/36) of all mutations. Among transitions, 76.5% (26/34) were C:G > T:A, whereas 23.5% (8/34) were A:T > G:C. Transversions accounted for only 5.6% (2/36) (all of them C:G > G:C) (Fig. 2B and Table S3). Important differences were found between BPS spectra of *M. tuberculosis* H37Rv and another Mycobacterium like *M. smegmatis* mc^2^ 155, when the spectrum was analyzed via mutations in the *rpoB* gene. In *M. smegmatis*, the spectrum is also biased toward transitions, although not so heavily as in H37Rv, with a 72.5%, with a 66.7% of C:G > T:A changes (4) (Fig. 2B). The transition/transversion (Tr/Tv) ratio in *M. tuberculosis* H37Rv is 17.0 (34/2) (Table S3), significantly higher than previously reported values for other Actinobacteria, including *M. smegmatis* (2.63) (4), *C. glutamicum* (2.3) (6), and *S. ambofaciens* (1.01) (13).

Deletion of *nucS* in *M. smegmatis* led to 100% transitions (4), with a shift toward A:T > G:C (from 33.3% in WT to 56% in the Δ*nucS* strain) and the consequent decrease in C:G > T:A (from 66.7% in WT to 44% in Δ*nucS*) (Fig. 2B). However, in *M. tuberculosis* Δ*nucS*, although transitions remained predominant (94.4%, 34/36), the spectrum shifted. C:G > T:A substitutions increased from 76.5% in WT to 97.1% (33/34) in Δ*nucS* (*P* = 5.121 × 10^−6^; unilateral binomial test), whereas A:T > G:C substitutions declined from 23.5% in WT to 5.6% (2/36) (Fig. 2B and Table S3). This suggests that, differently from *M. smegmatis*, NucS selectively prevents C:G > T:A substitutions in *M. tuberculosis*. Transversions remained unchanged, 5.6% in both Δ*nucS* and WT, and the Tr/Tv ratio in Δ*nucS* (17.0) was identical to WT. Interestingly, a high Tr/Tv ratio is a hallmark of MMR inactivation in Actinobacteria and other bacteria (see Discussion section). Therefore, the high Tr/Tv ratio in wild-type H37Rv (17.0) and its identical value upon *nucS* deletion suggest an inherently diminished MMR activity.

### Predicted effect of the H37Rv-specific R144S polymorphism on the structure of NucS

**Fig 3:**
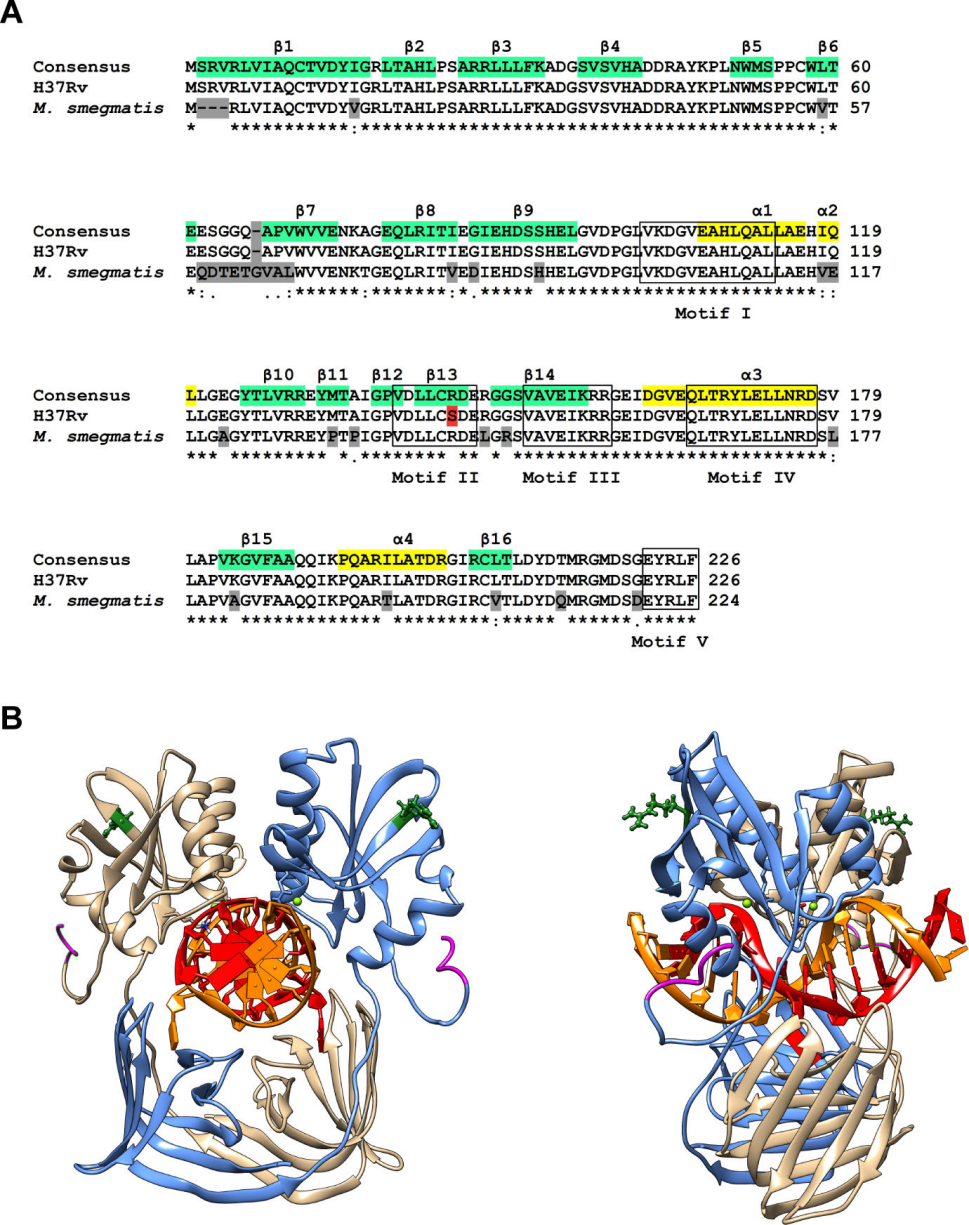
Sequence alignment and predicted structure of *M. tuberculosis* NucS-DNA complex. (**A**) The consensus sequence of NucS from *M. tuberculosis* (see Materials and Methods) is shown aligned against the sequences from *M. tuberculosis* H37Rv and *M. smegmatis* mc^2^155 using Clustal V. Differences have been highlighted in gray, except for variant R144S in *M. tuberculosis* H37Rv, which has been highlighted in red to ease its identification. The degree of NucS sequence conservation is indicated below the sequences using clustal conventions (*, totally conserved; “:,” similar chemical properties; “.,” weak conservation; blank, not conserved) (https://www.ebi.ac.uk/). Sequence motifs conserved across *Archaea* (29) are boxed and labeled on the bottom line (motif V corresponds to the C-terminal PCNA binding motif). Structural features are highlighted in yellow (alpha-helix) and turquoise (beta-sheet) over the reference *M. tuberculosis* consensus sequence and named in the top row of the alignment. Sequence numbering is indicated on the right side for each sequence. (**B**) Model of the complex of the *M. tuberculosis* NucS dimer bound to DNA, colored as in reference 8 to facilitate comparison (NucS monomers are colored blue and brown each, DNA chains red and orange). Two views are presented: a front view looking down DNA major axis, and a side view rotated 90°. Residue R144 is shown as a ball and stick model colored in forest green, and the β-clamp-binding motif in magenta. The DNA sequence contains a G:T mismatch. The catalytic domain is placed above the DNA in the figure, and the mismatched DNA-binding domain is placed below.

The high Tr/Tv ratio observed in *M. tuberculosis* H37Rv wild-type, coupled with the lack of a significant increase in mutation rate upon *nucS* deletion and the altered transition spectrum, suggests that the NucS activity may be partially impaired in this species. Notably, sequence analysis demonstrated that the H37Rv NucS deduced sequence (Rv1321) harbors a single amino acid substitution, arginine (R) to serine (S) at position 144 (R144S), compared with the *M. tuberculosis* NucS consensus sequence (26) (Fig. 3A). Moreover, a previous work (4) proposed that this polymorphism may contribute to an increased rate of spontaneous resistant mutations, prompting us to investigate whether it could be attributed to alterations in protein conformation.

To enable a comparison between wild-type and the mutant protein, it first required a structural model of *M. tuberculosis* NucS. Since no known experimental structure is currently available, we generated homology-based models of both apo (unbound) and DNA-Mg^2+^-bound dimeric forms for the R144 (wild-type) and S144 (mutant) variants. These models closely align with published structures and reveal that *M. tuberculosis* NucS, while exhibiting secondary structure differences relative to archaeal homologs, retains the overall fold and conserved motifs in both conformational states (Fig. 3B).

Residue 144 is located in region β-strand 13 of *M. tuberculosis* NucS C-terminal domain (corresponding to β 10 in *T. kodakarensis*) but lies outside motif V (29). Positioned on the complex outer surface and relatively distant from the C-terminal QLxxLF β-clamp binding motif (8) (EYRLF in *M. tuberculosis*), it is unlikely to directly participate in clamp binding (Fig. 3B). Its solvent-facing orientation and location outside core functional motifs further suggest that it plays no direct role in DNA binding, recognition, or dimerization. However, the substitution of arginine with serine at position 144 leads to changes in both charge distribution and surface topology. The solvent-accessible surface area (SASA) of this region decreases from 358 Å^2^ with R144 to 226 Å^2^ with S144, replacing a prominent, positively charged protrusion of arginine with a flatter, dipolar, and hydrophobic patch formed by serine (Fig. S2). These alterations suggest that residue 144 could influence interaction stability at molecular interfaces.

### Prevalence of the R144S polymorphism among clinical isolates and its relationship with resistance

**Fig 4:**
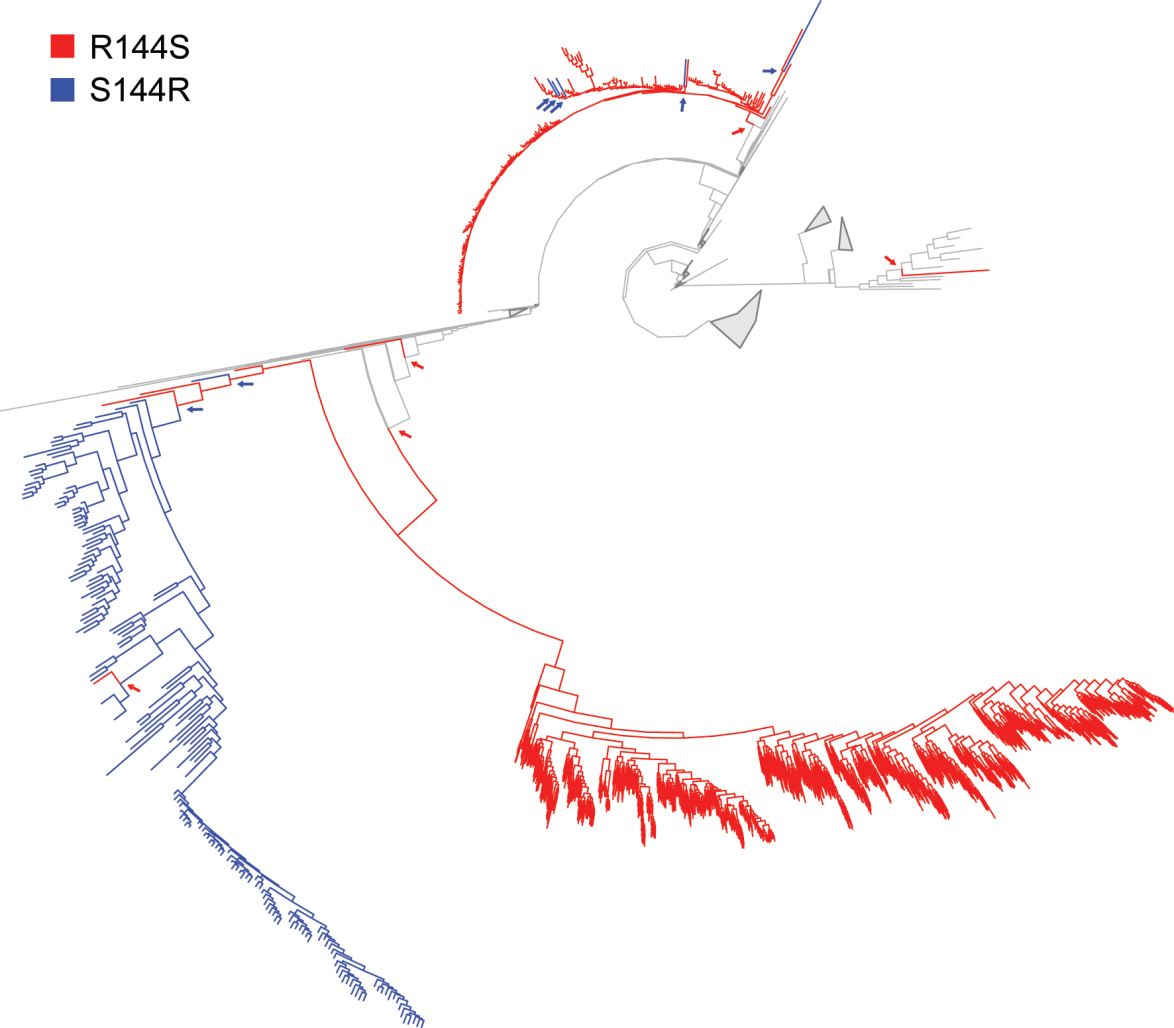
Phylogenetic subset tree (*n* = 7240) of lineage four showing the distribution of homoplasy associated with the R144S NucS mutation. The tree was subset from the most recent common ancestor (MRCA) of the phylogenetic branches showing homoplasy. Red branches and arrows indicate the independent propagation of the R144S change, whereas blue branches represent S144R reversions. Gray branches represent no homoplasy detected, displaying the original R144 sequence. Uninformative clades were collapsed (gray triangles), and the representative clades containing homoplastic events were rescaled to ease the visualization of the R144S SNP distribution across the phylogeny. The R144S mutation arose independently in three branches, two of which were classified under sub-lineage 4.9 (top and bottom colored branches). The right branch was identified under lineage 4.

To investigate the potential link between R144S and antibiotic resistance acquisition in *M. tuberculosis*, we screened 14,278 genomes from the Bacterial and Viral Bioinformatics Resource Center (BV-BRC) database, which includes experimentally determined antibiotic susceptibility profiles. Consistently, R144S remained the most common NucS polymorphism, identified in 79 strains (0.55%). We conducted χ^2^ and Fisher’s exact tests to assess the association between this polymorphism and resistance to 20 different antibiotics (ethambutol, kanamycin, delamanid, clofazimine, streptomycin, rifampicin, rifabutin, prothionamide, ofloxacin, linezolid, isoniazid, capreomycin, amikacin, moxifloxacin, ethionamide, pyrazinamide, para-aminosalicylic acid, nicotinamide, levofloxacin, and bedaquiline) for which data were available. Among these, the NucS R144S polymorphism showed a strong statistically significant association with ethambutol resistance, but not with other antibiotics. Specifically, 1.19% (44/3,702) of EMB-R strains carried the R144S polymorphism, compared with only 0.32% (35/10,912) of EMB-susceptible strains (*P* < 0.0001). This result suggested, in the absence of other data, a potential role for the R144S substitution in promoting ethambutol resistance. However, the BV-BRC data set is based on assembled genomes, which limits the ability to apply quality filtering steps typically used in analyses of raw paired-end short read data. To strengthen our findings, we analyzed a larger data set comprising 44,921 *M*. *tuberculosis* genomes with available raw whole genome sequencing (WGS) (30, 31) (Table S4). Within this data set, the R144S polymorphism was detected in 2.95% of strains (1,328/44,921), accounting for ~67.5% (1,328/1,965) of all *nucS* polymorphisms. Notably, almost all R144S-positive polymorphisms belonged to the Euro-American lineage 4 (~96%; 1,278/1,328), and more specifically to the H37Rv sub-lineage 4.9 (~94%; 1,251/1,328) (Fig. 4). However, within sub-lineage 4.9, which includes 5,008 strains in our data set, R144S is present in only ~25% of samples (1,251/5,008), indicating that it is not a sub-lineage-defining polymorphism.

We then assessed the association between R144S and the predicted genotypic resistance across the 44,921 strains. Of the 1,328 R144S-positive strains, 1,271 showed susceptibility to all the antibiotics screened, whereas 51 showed rifampicin resistance and six showed linezolid resistance. Fisher’s exact tests revealed a significant association between R144S and linezolid resistance, but none for rifampicin nor any other antibiotic of the 13 tested (amikacin, kanamycin, capreomycin, ethambutol, ethionamide, fluoroquinolones, isoniazid, ethionamide, linezolid, para-aminosalicylic acid, pyrazinamide, rifampicin, and streptomycin). However, given the low number of linezolid-resistant strains, this association may be spurious. To further investigate the potential selective pressure acting on the R144S polymorphism and its potential association with antibiotic resistance, we performed a genome-wide association study (GWAS) that accounts for the population structure of the data set. No significant association between R144S and antimicrobial resistance was detected, supporting the hypothesis that the observed link with linezolid resistance may be due to limited sample size.

We also examined the evolutionary constraints of *nucS* by analyzing the SNP density across the gene among the *M. tuberculosis* strains in our data set. Interestingly, *nucS* exhibited significantly fewer SNPs per kilobase per strain (0.0651) compared with the genome-wide average (0.1384; Poisson *P*-value < 0.00001). Moreover, *nucS* also showed significantly lower SNP density than other genes involved in DNA replication, repair, and recombination (3R genes; mean = 0.1195; Poisson *P*-value < 0.00001) based on a curated list of 39 3R genes from Zein-Eddine et al. (32). These findings suggest that *nucS* is under purifying selection.

Finally, to assess whether the R144S polymorphism arose independently during *M. tuberculosi*s evolution, we analyzed homoplasy at codon 144 across the phylogeny of the 44,921 strains. We identified homoplasy events at this codon: 5 R > S and 7 S > R changes (Fig. 4). These observations indicate signatures of parallel evolution at codon 144 within lineage 4, suggesting that the recurrent emergence of R144S might be driven by selective pressures.

### Effect of the change in amino acid 144 of NucS on antibiotic resistance and BPS spectra

To directly assess the functional impact of the NucS R144S polymorphism on the acquisition of resistance mutations, we introduced the S144R substitution (restoring the amino acid present in the consensus sequence) into the *M. tuberculosi*s mc^2^6230 and H37Rv strains via oligo-mediated recombineering, generating mc^2^6230-S144R and H37Rv-S144R, respectively.

**Fig 5:**
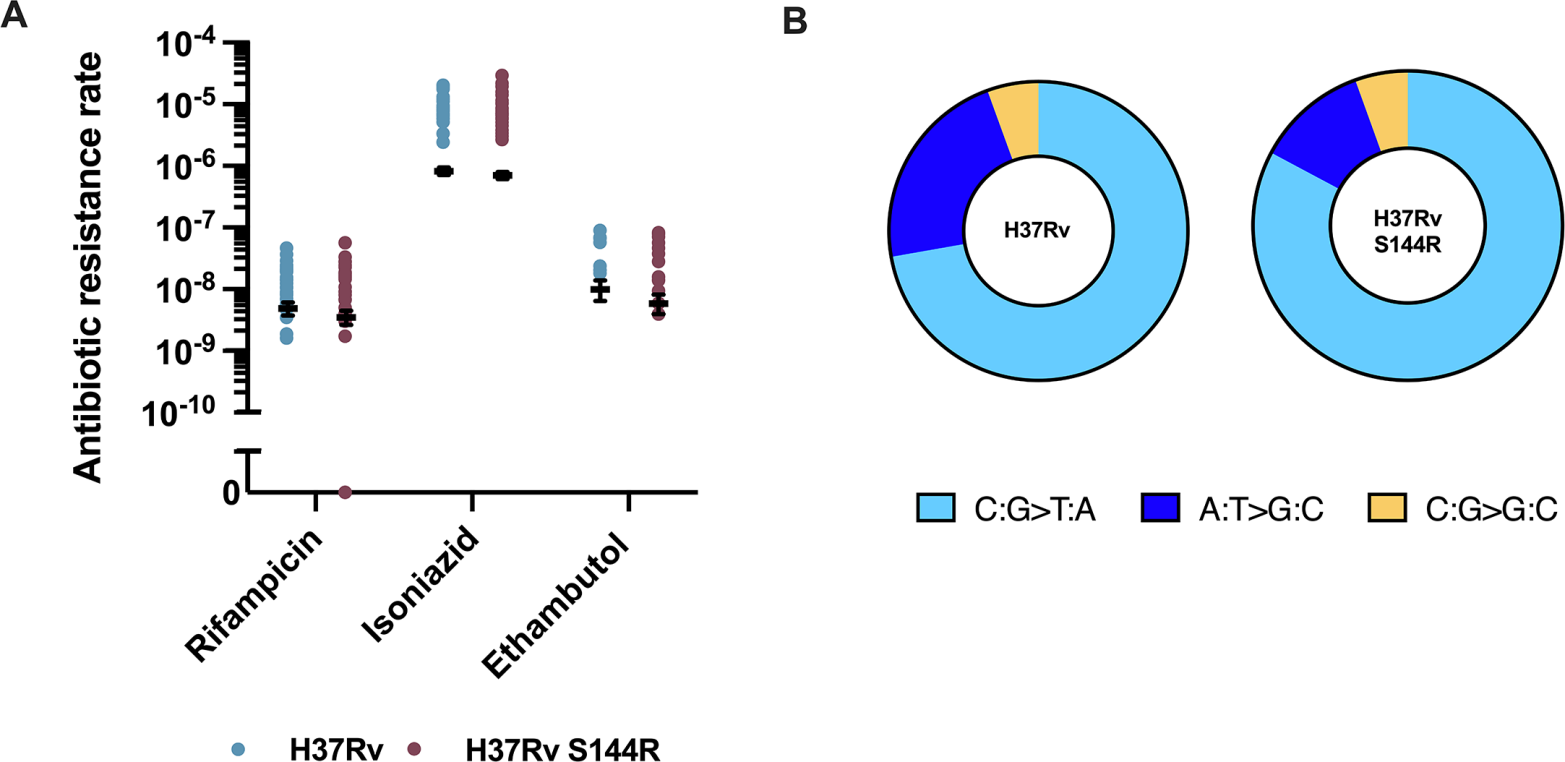
Effect of the change in amino acid 144 of NucS on antibiotic resistance and BPS spectrum. Rates of spontaneous RIF-R, INH-R, and EMB-R mutants in *M. tuberculosis* strains H37Rv and its S144R derivative (**A**) and mutational spectrum of these strains. (**B**) Spectra are expressed as percentage of RIF-R mutations. Transitions are shown in blue tones, transversions in orange tones. Between 1 and 5 colonies from each fluctuation test culture were pooled. Only colonies with different mutations from the same culture were considered for the study (Table S3).

In the mc^2^6230-S144R strain, the RIF-R resistance mutation rate was 3.04 × 10^−^⁹ (95% CI: 2.31–3.84 ×10^−^⁹), resulting in a small, although significant, 1.81-fold decrease when compared with the mc^2^6230 WT strain (Table S1). Fluctuation tests for resistance mutation rates to RIF-R, INH-R, and EMB-R in H37Rv-S144R yielded values of 3.34 × 10^−^⁹ (95% CI: 2.51–4.29 × 10^−^⁹), 6.91 × 10^−7^ (95% CI: 6.01–7.78 × 10^−7^), and 5.61 × 10^−^⁹ (95% CI: 3.75–7.83 × 10^−^⁹), respectively (Fig. 5A and Table S1). These findings indicate that although the S144R substitution slightly alters the rate of spontaneous resistant mutants when compared with wild-type strains, it does not account for the non-mutator phenotype observed for the Δ*nucS* derivatives. Therefore, although a statistically significant relationship between the R144S substitution and ethambutol resistance was observed by analyzing a genome sequence database, this association cannot be attributed to an increased mutation rate to EMB-R or differences in MIC of ethambutol (Table S2).

The mutational spectrum was only slightly changed upon restoring the consensus NucS sequence. Although the transitions remained predominant (94.4% transitions versus 5.6% transversions) and the Tr/Tv ratio stayed identical (17), the prevalence of C:G > T:A transitions increased from 76.5% (WT) to 88.2% (H37Rv-S144R) (*P* = 0.04021; one-sided binomial test), whereas A:T > G:C transitions decreased from 23.5% (WT) to 11.8% (H37Rv-S144R). These values are intermediate between the WT and the Δ*nucS* derivative (Fig. 5B and Table S3).

## DISCUSSION

The post-replicative mismatch repair system, which corrects mismatches that escape both base selection and 3ʹ−5ʹ proofreading activities of replicative DNA polymerase (3), is essential for DNA replication fidelity and the maintenance of genome stability. Traditionally, this repair was thought to be a highly conserved process mediated by members of the MutS and MutL protein families. However, the identification of NucS as a key player in genome stability in some Actinobacteria and Archaea reshaped this paradigm, establishing the existence of at least two distinct MMR pathways: the canonical MutSL system and an independently evolved NucS-dependent pathway (4). Loss or mutational inactivation of *nucS* results in a hypermutable phenotype in all Actinobacteria previously tested, underscoring its critical role in the evolution and adaptability of these industrially and clinically relevant species.

In *M. tuberculosis,* where drug resistance arises exclusively via chromosomal mutations (20), understanding the mutational landscape is, therefore, particularly relevant (21). Despite the well-established role of NucS in safeguarding genomic stability and limiting resistance acquisition in other mycobacteria, its specific function in *M. tuberculosis* remains unknown, likely due to technical challenges in generating a *nucS*-null mutant and concerns about managing a potentially dangerous hypermutable phenotype.

Here, we systematically evaluated the role of *nucS* in the acquisition of drug resistance in *M. tuberculosis*. Deletion of *nucS* has been achieved in both the model strain H37Rv and its attenuated derivative H37Rv mc^2^6230. Unexpectedly, the deletion only marginally influenced the rate at which spontaneous RIF-R mutations appear in the mc^2^6230 strain and had no effect in H37Rv, challenging the presumed role of *nucS* as a principal guardian of genome stability and a modulator of antibiotic resistance acquisition in *M. tuberculosis*. These results contrast sharply with previous findings in other Actinobacteria, where *nucS* deletion in *M. smegmatis* (4), *S. coelicolor* (4), *S. ambofaciens* (13), and *C. glutamicum* (5, 6, 28) caused a two-orders-of-magnitude increase in RIF-R spontaneous mutation rate (Fig. 1B). This non-mutator phenotype also extended to resistance acquisition to other front-line antitubercular drugs, such as INH and EMB, with only a minimal (<2-fold) increase in the INH-R mutation rate upon *nucS* deletion in H37Rv. These observations suggest the existence of additional mechanisms beyond *nucS* that control spontaneous mutagenesis in *M. tuberculosis*, warranting further investigation.

Mutational spectrum analysis provides further evidence that *nucS* functions differently in *M. tuberculosis* compared with other Actinobacteria, consistent with previous findings of G-G mismatch repair activity in H37Rv (11). Wild-type H37Rv exhibits a high transition-biased mutation spectrum (94.4% transitions; Tr/Tv ratio of 17.0), markedly higher than ratios observed in other Actinobacteria (2.63 in *M. smegmatis* [4], 2.3 in *C. glutamicum* [6], and 1.01 in *S. ambofaciens* [13]). Notably, *nucS* deletion did not affect these parameters (transitions 94.4%; Tr/Tv ratio of 17.0), in contrast to other bacteria where MMR inactivation increases these ratios dramatically: *M. smegmatis* (38-fold) (14), *C. glutamicum* (44.6-fold) (6), and *S. ambofaciens* (36-fold) (13). Similarly, inactivation of the *mutSL*-based MMR led to Tr/Tv ratio increases in *E. coli* (33-fold) (33), *Pseudomonas fluorescens* (45-fold) (34), *Deinococcus radiodurans* (9-fold) (34), *Vibrio cholerae* (29-fold) (35), and *Vibrio fisheri* (102-fold) (35). The consistently high basal Tr/Tv ratio (17.0) of the wild-type H37Rv strain and its stability upon *nucS* deletion suggest that the *nucS*-dependent MMR pathway may already be impaired, despite a baseline RIF-R mutation rate comparable with other Mycobacteria, including *M. smegmatis* and *M. tuberculosis* strains belonging to the same lineage (4, 21).

Interestingly, the H37Rv NucS sequence carries a single R144S change relative to the *M. tuberculosis* NucS consensus sequence (26). Previous work proposed that this polymorphism may impair NucS function, leading to a hypermutable phenotype (4). Our structural model of *M. tuberculosis* NucS suggests that the R144S variant alters protein surface, potentially disrupting protein-protein interactions critical for mismatch repair function. In this sense, a STRING (36) database analysis identified several potential interaction partners, some of them related to DNA repair, which could be influenced by the R144S substitution. They include AlkA (DNA-repair enzyme involved in the adaptive response to alkylation damage in DNA), DnaN (DNA polymerase III β-chain, β-clamp), Ogt (Methylated-DNA-protein-cysteine methyltransferase; involved in the cellular defense against the biological effects of O6-methylguanine and O4-methylthymine in DNA), RpoZ (DNA-directed RNA polymerase subunit omega), RpsT (30S ribosomal protein S20), WhiB1, and WhiB2 (transcriptional regulatory proteins). Uncharacterized proteins include Rv1312, Rv1318c (possible adenylate cyclase), Rv1322, Rv1830, Rv2413c, Rv2738c, and Rv3114 (possible cytosine deaminase) (Fig. S3). These associations are meant to be specific and meaningful, that is, proteins jointly contribute to a shared function, although it does not necessarily mean they are physically binding to each other. However, the direct interaction of NucS with potential partners and the possible effect of the R144S mutation on these interactions remains to be assessed.

Initially, our bioinformatic screening of a genomic assemblies data set suggested a possible correlation between the R144S polymorphism and phenotypic ethambutol resistance. However, a GWAS analysis of a WGS data set of 44,921 *M*. *tuberculosis* genomes revealed no significant association between R144S and known antibiotic resistance mutations. These findings are consistent with our experimental data: fluctuation tests assessing RIF-R, INH-R, and EMB-R mutation rates in engineered strains expressing the restored consensus NucS sequence (S144R) showed only minimal differences in spontaneous resistance acquisition. Furthermore, the subtle changes observed in the mutational spectrum of H37Rv-S144R and the modest shifts in mutation rates do not account for the exceptionally high Tr/Tv ratio in H37Rv, nor do they explain the normo-mutator phenotype of the ∆*nucS* strain.

Genomic analysis revealed that *nucS* might be under strong purifying selection, aligning with previous results that describe an evolutionary trajectory of positive selection toward the ancestor, followed by purifying selection toward terminal branches (31). The R144S *nucS* polymorphism, although predominantly found within sub-lineage 4.9, exhibits recurrent homoplasy within lineage 4, suggesting that it may have arisen independently. This pattern points to a non-neutral evolutionary role of the R144S change, possibly shaped by sub-lineage-specific selective pressures.

In conclusion, although *nucS* is central to maintaining genome stability and low mutation rates in other Actinobacteria, its impact on mutagenesis in *M. tuberculosis* appears limited. The persistent low mutation rate in H37Rv, even following *nucS* deletion or alteration, points to additional, yet unidentified, mechanisms governing post-replicative DNA repair and evolutionary adaptation in this pathogen. Dissecting the interplay between DNA repair pathways, mutation dynamics, and antibiotic resistance in *M. tuberculosis* will be critical to inform future therapeutic and diagnostic strategies in the ongoing battle against tuberculosis.

## MATERIALS AND METHODS

### Bacterial strains, culture conditions, oligonucleotides, and media

The *M. tuberculosis* strains H37Rv and H37Rv mc^2^6230 (attenuated strain from the Jacobs laboratory at Albert Einstein College of Medicine, genotypically identical to H37Rv mc^2^6030 (27), except it does not contain a hygromycin resistance marker) were obtained from W. Jacobs laboratory. Strains and their mutant derivatives used in this work are listed in Table S5. All oligonucleotides used in this work are listed in Table S6.

*M. tuberculosis* H37Rv liquid cultures were grown on Middlebrook 7H9 (Becton Dickinson) supplemented with 0.5% glycerol, 0.05% Tween 80, 10% oleic acid, albumin, dextrose, and catalase (OADC) (Becton Dickinson), and incubated at 37°C for 7–15 days. For solid media, Middlebrook 7H10 (Becton Dickinson) supplemented with 0.5% glycerol, 0.05% Tween 80, 10% OADC, and 500 ng/mL amphotericin B (CAS No 1397-89-3, NeoBiotech) was used, and plates were incubated at 37°C for 3–4 weeks.

When working with *M. tuberculosis* H37Rv mc^2^6230 auxotrophic strain, all media were supplemented with 24 µg/mL D-pantothenic acid hemicalcium salt (CAS No 137-08-6, Merck) and 0.2% OmniPur Casamino Acid (CAS No 65072, Calbiochem).

Media was supplemented with antibiotics when appropriate: 25 μg/mL kanamycin (Gibco), 20 μg/mL streptomycin (CAS No 3810-74-0, Merck), 50 μg/mL hygromycin (CAS No 31282-04-9, Sigma-Aldrich), 4 μg/mL rifampicin (Rifaldin, Sanofi), 2 μg/mL isoniazid (CAS No 54-85-3, Merck), 5 μg/mL thiostrepton (CAS No 1393-48-2, Calbiochem), and 10 μg/mL ethambutol (CAS No 1070-11-7, Merck).

Cloning was performed in *E. coli* DH5α grown in LB medium supplemented with 50 μg/mL kanamycin or 12.5 μg/mL chloramphenicol (CAS No 56-75-7, Nzytech) when appropriate.

### Construction of deletion mutants

*M. tuberculosis* H37Rv mc^2^6230 and H37Rv Δ*nucS* (Rv1321) in-frame deletion mutants were generated using the ORBIT technology (37). RecT was induced ~16 h before transformation by the addition of ATc to a final concentration of 500 ng/mL. Competent *M. tuberculosis* mc^2^6230 and H37Rv strains carrying plasmid pKM461 were transformed with 1 μg of *nucS* ORBIT oligonucleotide (Table S5) and 200 ng of pKM464. Cells were recovered in 10 mL of 7H9 and grown for 24 h at 37°C. After outgrowth, cells were plated on 7H10 plates supplemented with hygromycin. Putative deletion colonies were confirmed by PCR with oligos nucS_CDS_FW and nucS_CDS_RV; ORBIT_nucS_FW 5′-oriE Rv to verify the absence of the *nucS* gene and the 5’ junction, respectively. Additionally, the mutants were confirmed by WGS to discard the presence of additional relevant mutations in the genome.

### Complementation of Δ*nucS* mutants

For complementation, the *M. tuberculosis* Δ*nucS* mutants, the wild-type *nucS* sequence from H37Rv, were cloned into the backbone of integrative vector pMV361 (38). The *nucS* coding sequence and 479 bp of its upstream region were PCR amplified from genomic DNA of *M. tuberculosis* H37Rv, using oligos new-nucS_EcoRI_F and nucS_TB_HindIII_R (Table S6). Plasmid pMV361 was PCR amplified with oligos pMV_KO_Hsp60_Eco_R and pMV_Hsp60_HindIII_F. The PCR amplification products were digested with restriction enzymes EcoRI and HindIII (New England Biolabs) and ligated using T4 ligase to eliminate the p*hsp60* gene promoter.

The original *aph* kanamycin resistance gene from plasmid pMV361 was replaced by a chloramphenicol resistance cassette (Cam^R^). The (Cam*^R^*) was amplified by PCR from the pKM464 plasmid (37) using the primers cam gene FOR and cam gene REV (Table S6) and inserted into the plasmid carrying *nucS* previously digested with *Nhe*I-*Ase*I using the Gibson technology, obtaining the pMV361 *nucS* H37Rv Cam^R^ plasmid.

Finally, a thiostrepton resistance cassette (Tsr^R^) was amplified from pSETtsr (39) using primers tsr gene FOR and tsr gene REV (Table S6) and inserted into the pMV361 *nucS* H37Rv Cam^R^ plasmid previously digested with *Kpn*I and *Not*I enzymes using the Gibson technology, obtaining the pMV361 *nucS* H37Rv plasmid. The putative complemented mutant was obtained upon electroporation of the pMV361 *nucS* H37Rv plasmid into the *M. tuberculosis* H37Rv Δ*nucS* strain and incubation of the plated samples on Middlebrook 7H10 agar plus thiostrepton for 3 weeks at 37°C. Finally, the *M. tuberculosis* H37Rv Δ*nucS* complemented mutants were analyzed by PCR and Sanger sequencing.

### Identification of *nucS* 144 SNP in *M. tuberculosis* databases and association with phenotypic resistance

To identify the NucS consensus sequence in *M. tuberculosis,* we extracted all cognate sequences from the NCBI (https://www.ncbi.nlm.nih.gov/), aligning them with Clustal Omega (https://www.ebi.ac.uk/jdispatcher/msa/clustalo) and identifying the consensus with Jalview (40). Average conservation was 99.5%.

To analyze whether the R144S mutation of NucS of *M. tuberculosis* was associated with AMR phenotypes, the distribution of the polymorphism in a database with AMR phenotypic information was first studied. The *M. tuberculosis* genomes from the strains with experimentally determined resistance phenotype data were retrieved from the Bacterial and Viral Bioinformatics Resource Center database (BV-BRC; www.bv-brc.org, accessed on 02-11-2025; *n* = 14,278). The presence of R144S was assessed by comparing each genome against the *nucS* gene using Snippy v.4.6.0. The *nucS* sequence from *M. tuberculosis* H37Rv, which carries the R144S mutation (RefSeq NP_215837.1), was employed as a reference. The statistical significance of the association between the presence of the R144S mutation and the AMR phenotype to multiple antibiotics (*n* = 20) was assessed by χ^2^ tests (or Fisher’s exact test if *n* < 5 in the contingency table). *P* values were adjusted for multiple comparison testing by the Bonferroni-Holm method.

### GWAS approach to test for genotype-phenotype association of *nucS* mutations

We analyzed the WGS data from 44,921 samples available (30, 41) (Table S4) using fasterq-dump (https://trace.ncbi.nlm.nih.gov/Traces/sra/sra.cgi?view=software and the SRA Toolkit Development Team) by directly accessing the Sequence Read Archive (SRA) of the National Center for Biotechnology Information (NCBI). We used Snippy (https://github.com/tseemann/snippy) to call variants against the inferred common ancestor genome sequence of *Mycobacterium tuberculosis* (42), which does not carry the R144S SNP, contrary to the commonly utilized H37Rv strain as reference. We then annotated the VCF files using SnpEff (43) and filtered the results using bcftools (44). Specifically, we removed the repetitive regions and kept high-quality biallelic SNPs (QUAL > 100 & min. read depth = 10), resembling the parameters of commonly utilized tools which employ the *M. tuberculosis* H37Rv strain as reference, such as TB-annotator (45). We classified the samples into their respective lineage using a curated list of SNPs based on comparisons with the common inferred ancestor. These results were cross-validated with lineage assignments reported by TB-Profiler (46). Similarly, genotypic resistance was inferred using a dedicated SNP list aligned to the same ancestral reference, and the predictions were supported by resistance profiles from TB-Profiler. Both SNP lists were extracted from the publicly available repository of the Tuberculosis Genomics Unit at the IBV-CSIC (TGU file repository; https://gitlab.com/tbgenomicsunit/ThePipeline). We reconstructed each strain genomic sequence based on the high-quality SNPs called using bcftools (44) consensus and executed snp-sites (47) over the whole genomic sequences to obtain the core SNPs alignment. This was used as input for FastTreeML (specifying -gtr and -gamma parameters) (48), which allowed us to infer the phylogeny of the 44,921 strains. The genotype obtained with Snippy, together with the phylogeny and the genotypic predicted AMR phenotypes, was used as input for performing the GWAS analysis of *nucS* using TreeWAS (49) as done in Zein-Eddine et al. (32).

### SNP accumulation in *nucS*

To measure the SNP burden over *nucS*, we computed the raw SNP counts normalized by gene length over the 44,921 genomes and fitted a Poisson model to test for the possible increase of mutations in *nucS*. We calculated the density of SNPs per kilobase pair (kbp) per strain in each gene and performed an enrichment test fitting a Poisson distribution. We then compared the density of SNPs per strain in *nucS* with the rest of the genes over the *M. tuberculosis* genome, as well as with a subset of genes involved in DNA repair (3R genes) (32).

### Homoplasy detection

To analyze the possible homoplasy of the R144S SNP, we ran SNPPar (50) with default parameters. We used the phylogenetic tree inferred with FastTreeML (48) and the annotated ancestral reference GBK (30, 31). We included the Snippy results to provide the R144S SNP distribution across the phylogeny.

### Construction of strains mc^2^6230-S144R and H37Rv-S144R and validation

NucS S144R mutant derivative of *M. tuberculosis* H37Rv was generated through mycobacterial oligo-mediated recombineering as previously described (51), with some modifications. In brief, 70-mer oligonucleotides were designed to correspond to the lagging strand of the replication fork at the *nucS* gene genomic region, with the desired mutation in the middle of the oligo. RecT expression was induced ~16 h before transformation by the addition of ATc to a final concentration of 500 ng/mL. 400 μL of *M. tuberculosis* H37Rv or mc^2^6230 carrying pKM461 competent cells were transformed with 2.5 µg of nucSTB_S144R oligo and 0.1 µg of rpsL_K43R_TB oligo (Table S5). The rpsL_K43R_TB oligo generates streptomycin resistance by generating a K43R mutation into the *rpsL* gene in the bacterial chromosome (51). Cells were recovered in 5 mL of 7H9 supplemented with kanamycin. After 3 days of recovery, a 1:25 dilution of the cultures was performed in 7H9 supplemented with streptomycin. This culture was grown until OD_600_ = 1 (approximately 28 days) at 37°C, and 500 μL of a 10^−6^ dilution was plated into 7H10 plates supplemented with streptomycin. The strains were cured of the pKM461 plasmid by selecting individual clones growing in 7H10 plates supplemented with 3% sucrose (52). Individual colonies were picked and screened for the presence of the SNP in the *nucS* gene through PCR amplification with oligos Seq_1F_nucS_TB and Seq_1R_nucS_TB (Table S5). The PCR-amplified products were purified following the manufacturer’s instructions using QIAquick PCR Purification Kit (Qiagen) and were Sanger sequenced (StabVida) with oligo Seq_2F_nucS_TB (Table S5) to verify the mutant genotype. Additionally, the *M. tuberculosis* H37Rv mutants were confirmed by WGS (see below).

### Whole genome sequencing and strain validation

To check and verify the constructed strains, genomic DNA was extracted following the standard protocol for preparation of high-quality mycobacterial genomic DNA (53). The integrity of each DNA sample and the absence of RNA contamination were confirmed by DNA agarose gel electrophoresis, whereas its concentration and purity were measured using a NanoDrop-2000 spectrophotometer (Thermo Fisher Scientific). Genomes were sequenced through Illumina MiSeq sequencing using a MiSeq v.2 sequencing kit to obtain 250-bp paired-end reads (StabVida). The sequences were compared with those of their wild-type strain to verify the absence of other potential mutagenic SNPs.

Paired reads were assembled against the published reference genome NC_000962.3 using UGENEv52.0 (54). Raw DNAseq protocol, which encompasses read quality control with FastQC (https://www.bioinformatics.babraham.ac.uk/projects/fastqc/), quality trimming and adapter removal with CutAdapt (55), read assembly with BWA-MEM (56), and post-processing with samtools/bcftools (44) using the default parameters.

Variants with respect to the published reference genome were detected, split into SNPs and indels, and filtered (QD < 2, FS > 60, MQ < 40, SOR > 10 for SNPs, and QD < 0, FS > 200, SOR > 10 for indels) using the aligned reads and a standard best-practices protocol using GATK (57).

Identified variants were compared using bcftools to tell strain-specific from shared variants, annotated with snpEff (58), to highlight high-impact and loss-of-function variants, and further annotated from the GTF reference annotation.

### MIC determinations

Microtiter plates to calculate minimal inhibitory concentrations (MICs) were prepared by serially diluting 4× stocks of rifampicin (0.128 µg/mL–0.00025 µg/mL) and isoniazid (4 µg/mL–0.008 µg/mL) with 7H9 on sterile, clear, round-bottomed, 96-well microplates (Thermo Scientific Nunc MicroWell). After outgrowth on 7H9, 1:200 cell suspensions were prepared for *M. tuberculosis* mc^2^6230, H37Rv, and their *nucS* mutant derivatives. Plates were inoculated with 100 µL of the cell suspension. To prevent evaporation, 200 μL distilled H_2_O was added to the outer perimeter wells. Each plate included a negative control and a growth control. Plates were covered with their lids, placed in individual plastic bags, and incubated at 37°C for 6 days. After incubation, 30 μL of 100 μg/mL resazurin sodium salt solution (CAS-No: R7017-1G, Sigma-Aldrich) was added to each well and further incubated for 48 h at 37°C. MICs were assessed using a microplate reader with 555 nm excitation and 590 nm emission (FLUOstar Omega). Each MIC had three technical replicates. In the case of ethambutol, MIC was evaluated in solid medium. Culture replicates of H37Rv and its mutant derivatives were grown until OD_600_ = 1. Finally, 5 μL of the different strains was added to plates containing different concentrations of ethambutol (0, 1, 2, 4, 8, and 16 μg/mL), and growth was checked after 3 weeks.

### Bacterial growth

Plasmid pYUB3054 is a plasmid carrying GenL, a *M. tuberculosis* codon-optimized nanoluciferase (nluc*_M. tuberculosis_*) (59) amplified from the Addgene plasmid #85200, the integration system of phage tweety (60), and an *aph* kanamycin resistance cassette.

Plasmid pYUB3054 was transformed into *M. tuberculosis* H37Rv and its *nucS* mutant derivatives. Bacterial growth was evaluated by growth curve assays of *M. tuberculosis* H37Rv and its *nucS* mutant derivatives and using the correlation between relative luminescent units (RLUs) and viable cells. Four independent 10 mL cultures starting at OD_600_ 0.05 were incubated at 37°C in static condition, and nanoluciferase activity was measured every 24 h for a total of 18 days. RLUs were evaluated on white, 96-well microplates (Ref 655073, Greiner Bio-One) and measuring nanoluciferase activity. The Nano-Glo Luciferase Assay System (Promega) was used following the manufacturer’s instructions. Briefly, the Nano-Glo substrate was diluted 1:50 in Nano-Glo buffer and mixed 1:1 (vol/vol) with the samples. After the addition of substrate, plates were covered with adhesive, optically clear plate sealer (Microseal B PCR plate-sealing film, adhesive, optical no. MSB1001; Bio-Rad) and decontaminated, and luminescence was read immediately on a FLUOstar Omega spectrophotometer.

Growth curves were compared using a Baranyi model fitted using R (61) package. Growth rates and, to avoid biases due to experimental conditions, μ-max (maximum growth rate) values were checked for normality with Shapiro-Wilk test and for homoscedasticity with Bartlett’s test, and differences were compared using ANOVA followed by Tukey *post hoc* tests.

### Estimation of mutation rates

Mutation rates of *M. tuberculosis* H37Rv mc^2^6230, H37Rv, and their *nucS* mutant derivatives to each antibiotic were determined by fluctuation tests and validated by up to four different experiments each. Briefly, an exponential phase culture (1–5 × 10^7^ cells/mL) of each strain was diluted 1:1,000 to generate eight independent liquid cultures in Middlebrook 7H9 broth and incubated 15 days at 37°C. All grown cultures (1–2 × 10^8^ cells/mL), and serial dilutions were plated on Middlebrook 7H10 agar plates without antibiotic (for estimation of viable cells) and with antibiotic (for drug-resistant cells) and incubated for 28 days at 37°C. After this incubation, CFUs were counted to obtain the total number of viable cells (Nt) and mutant cells in the cultures.

To estimate the mutation rate (μ), data were pooled from multiple fluctuation test experiments as previously described by Zheng (62). Briefly, the mutation rate (μ) was recast as a function of the parameter β, defined by μ = e^β^. In this way, the maximum likelihood estimate (MLE) of β was found using the optimize function in R, and the 95% confidence intervals (CIs) for β were constructed based on the likelihood ratio principle using the R function uniroot.

The 99% confidence intervals were constructed as previously described and were used exclusively to compare mutation rates at the 0.001 significance levels (63). This approach was chosen to address the non-standard problem of pooling data from different fluctuation test experiments. As described by Zheng (64), using overlapping confidence intervals is particularly suitable for comparing mutation rates when experiments involve varying terminal population sizes, ensuring reliable statistical conclusions without simplifying assumptions.

### Mutational spectra

The mutational spectra of *M. tuberculosis* strains H37Rv and its mutant *nucS* derivatives were characterized by picking individual resistant colonies from RIF plates. Between 1 and 5 colonies per plate were isolated, and colonies from all the fluctuation tests were pooled, allowing for more than 40 colonies per strain. To avoid estimating mutations from the same original mutant, only different mutations from the same culture were considered for the study of the mutational spectrum.

For resistance to rifampicin, the rifampicin resistance-determining region in the *rpoB* gene was PCR amplified using a pair of primers RifRRDR-H37Rv Fw and RifRRDR-H37Rv Rv (Table S3). PCR products were sequenced using oligo RifRRDR-H37RvFW and compared with the reference gene sequences to identify the type of mutation in each RIF-resistant isolate.

Tr/Tv rates were compared using a log likelihood ratio test and differences in spectra were compared using a proportions test with Yate’s continuity correction.

### Protein structure prediction

We used the protein sequence of *M. tuberculosis* strain CD1551 NucS from entry P59979.1 (perfectly matching the NucS consensus sequence) to build a full-sequence 3D structural model using I-TASSER (65). To model the dimer, we next used as reference the published structures 5GKE, 5GKF, 5GKG, 5GKH, 5GKI, and 5GKJ (8) from PDB (66). After selecting the best-matching protein model, we superposed two CD1551 subunits over the available dimers, both in the apo and DNA-bound forms, then optimized the structures using the Amber force field, checked the results for conflicts, and selected the best models using UCSF Chimera (67). The DNA-Mg^2+^-bound dimer was then used to model the structure of the S144 variant using Modeler (68) with a very large refinement. The predicted structure was subjected to additional minimization using Amber and inspected in UCSF Chimera.

## Data Availability

All data supporting the findings of this study are available within the paper and its Supplementary Information. Raw WGS data were deposited into the NCBI Sequence Read Archive (SRA) under accession code PRJNA1277151 and will be publicly available after publication.

## SUPPLEMENTAL MATERIAL

Supplemental Material mbio.03310-25-s0001.docx Figures S1 to S3 and Tables S1 to S3, S5, and S6.

Table S4 mbio.03310-25-s0002.xlsx SRA IDs and origin of the *M. tuberculosis* genomes composing the data set.

ASM does not own the copyrights to Supplemental Material that may be linked to, or accessed through, an article. The authors have granted ASM a non-exclusive, world-wide license to publish the Supplemental Material files. Please contact the corresponding author directly for reuse.
