# Supplementary material for: The unique role of nucS-mediated noncanonical mismatch repair in Mycobacterium tuberculosis resistance evolution
Source: mBio. 2025 Dec 22;17(2):e03310-25. doi: 10.1128/mbio.03310-25 (PMC12892979; doi:10.1128/mbio.03310-25)
Supplement: Supplemental Material — Figures S1 to S3 and Tables S1 to S3, S5, and S6. [file mbio.03310-25-s0001.docx]

**Supplementary Material from** **“The unique role of *nucS*-mediated non-canonical mismatch repair in *Mycobacterium tuberculosis* resistance evolution” by Isabel Martín-Blecua et al.**

**Supplementary Tables.**

**Supplementary Table 1.** Mutation rates (**µ**, mutations/cell/generation) calculated from fluctuation tests for RIF-R, INH-R and EMB-R. Number of replicates (n) per strain and antibiotic, 95% confidence interval (CI) and fold increase compared to each respective wild-type strain (set to 1.00). Three stars: p < 0.001.

| **Strain** |  | **RIFAMPICIN** | | | |  | **ISONIAZID** | | | |  | **ETHAMBUTOL** | | | |
| --- | --- | --- | --- | --- | --- | --- | --- | --- | --- | --- | --- | --- | --- | --- | --- |
|  |  | **n** | **µ** | **CI 95%** | **Fold increase** |  | **n** | **µ** | **CI 95%** | **Fold increase** |  | **n** | **µ** | **CI 95%** | **Fold increase** |
| mc^2^6230 |  | 51 | 5.49 ×10^-9^ | (4.65-6.37) ×10^-9^ | 1 |  |  |  |  |  |  |  |  |  |  |
| mc^2^6230 Δ*nucS* |  | 26 | 1.55 ×10^-8^ | (1.24-1.89) ×10^-8^ | 2.83*** |  |  |  |  |  |  |  |  |  |  |
| mc^2^6230 S144R |  | 32 | 3.04 ×10^-9^ | (2.31-3.84) ×10^-9^ | 0.55*** |  |  |  |  |  |  |  |  |  |  |
| H37Rv |  | 32 | 4.66 ×10^-9^ | (3.58-5.86) ×10^-9^ | 1 |  | 23 | 8.09 ×10^-7^ | (6.95-9.20) ×10^-7^ | 1 |  | 8 | 9.57 ×10^-9^ | (6.20-13.30) ×10^-9^ | 1 |
| H37Rv Δ*nucS* |  | 16 | 5.88 ×10^-9^ | (4.18-7.79) ×10^-9^ | 1.26 |  | 20 | 1.57 ×10^-6^ | (1.37-1.76) ×10^-6^ | 1.94 *** |  | 16 | 6.88 ×10^-9^ | (4.10-10.40) ×10^-9^ | 0.72 |
| H37Rv Δ*nucS/nucS* |  | 8 | 3.33 ×10^-9^ | (1.82-5.29) ×10^-9^ | 0.71 |  | 8 | 9.96 ×10^-7^ | (7.89-11.9 x10^-7^) | 1.23 |  | 8 | 1.42 ×10^-9^ | (0.35-3.67) ×10^-9^ | 0.15 *** |
| H37Rv S144R |  | 32 | 3.34 ×10^-9^ | (2.51-4.29) ×10^-9^ | 0.72 |  | 24 | 6.91 ×10^-7^ | (6.01-7.78) ×10^-7^ | 0.85 |  | 16 | 5.61 ×10^-9^ | (3.75-7.83) ×10^-9^ | 0.59 |

**Supplementary Table 2.** MICs of antibiotics for strains.

| **Strain** | **RIF MIC** | **INH MIC** | **EMB MIC*** |
| --- | --- | --- | --- |
| mc^2^6230 | 0.004 |  |  |
| mc^2^6230 Δ*nucS* | 0.004 |  |  |
| mc^2^6230 S144R | 0.004 |  |  |
| H37Rv | 0.008 | 0.125 | 4 |
| H37Rv Δ*nucS* | 0.008 | 0.125 | 4 |
| H37Rv *nucS/nucS* | 0.004 | 0.125 | 4 |
| H37Rv-R144S | 0.008 | 0.125 | 4 |

* MIC of ethambutol was calculated using agar plates.

**Supplementary Table 3.** Mutational spectrum of strains H37Rv wild-type and its derivatives H37Rv-Δ*nucS* and H37Rv-S144R as analysed from sequences of *rpoB* of RIF-R mutants. 30 to 51 colonies from each strain were analysed yet only those containing different mutations from the same culture were considered for the study of mutational spectrum.

| **Position** | **Codon change** | **Amino acid change** | **H37Rv** | **H37Rv Δ*nucS*** | **H37Rv-S144R** |
| --- | --- | --- | --- | --- | --- |
| C 1322 G **(**Tv) | T**C**G→T**G**G | Ser 441 Trp | 1 |  |  |
| C 1322 T (Tr) | T**C**G→T**T**G | Ser 441 Leu | 1 |  |  |
| C 1333 T (Tr) | **C**AC→**T**AC | Hys 445 Tyr | 8 | 16 | 18 |
| C 1333 G (Tv) | **C**AC→**G**AC | Hys 445 Asp | 1 | 1 | 1 |
| A 1334 G (Tr) | C**A**C→C**G**C | Hys 445 Arg | 8 | 1 | 4 |
| A 1334 C (Tv) | C**A**C→C**C**C | Hys 445 Pro |  |  |  |
| A 1336 C (Tv) | **A**AG→**C**AG | Lys 446 Gln |  |  |  |
| C 1349 T (Tr) | T**C**G → T**T**G | Ser 450 Leu | 17 | 17 | 12 |
| C 1349 G (Tv) | T**C**G → T**G**G | Ser 450 Trp |  | 1 | 1 |
| Colonies analysed |  |  | 44 | 63 | 42 |
| **TOTAL BPS** |  |  | **36** | **36** | **36** |
| **% Transitions/BPS** |  |  | **94.4%** (34/36) | **94.4%** (34/36) | **94.4%** (34/36) |
| % C:G>T:A |  |  | 76.5% (26/34) | 97.1% (33/34) | 88.2% (30/34) |
| % A:T>G:C |  |  | 23.5% (8/34) | 2.9% (1/34) | 11.8% (4/34) |
| **% Transversions/BPS** |  |  | **5.6%** (2/36) | **5.6%** (2/36) | **5.6%** (2/36) |
| % C:G>G:C |  |  | 100% (2/2) | 100% (2/2) | 100% (2/2) |
| % A:T>C:G |  |  | **0%** | **0%** | 0% |
| Tr/Tv ratio |  |  | **17** | **17** | **17** |

**Supplementary Table 5.** Bacterial strains and their mutant derivatives.

| **Strain name** | **Genotype** | **Description** | **Markers** | **Source** |
| --- | --- | --- | --- | --- |
| H37Rv mc^2^6230 | ΔRD1 Δ*panC*D | Attenuated strain; auxotrophic for pantothenate | None | W. R. Jacobs |
| H37Rv | wild type |  | None | W. R. Jacobs |
| H37Rv mc^2^6230 Δ*nucS* | Δ*nucS*::pKM464 | mc^2^ 6230 deleted of Rv1321 by ORBIT | Hyg^R^ | This study |
| H37Rv Δ*nucS* | Δ*nucS*::pKM464 | H37Rv deleted of Rv1321 by ORBIT | Hyg^R^ | This study |
| H37Rv Δ*nucS* / nucS | H37Rv Δ*nucS* complemented with Rv1321 gene | H37Rv Δ*nucS*; pMV361 *nucS* H37Rv integrated at *attB* site (tRNA^Gly^) | Hyg^R^Cam^R^ Tsr^R^ | This study |
| H37Rv mc^2^6230-S144R | *nucS* S144R | Mutant generated by oligo-mediated recombineering | None | This study |
| H37Rv-S144R | *nucS* S144R | Mutant generated by oligo-mediated recombineering | None | This study |

**Supplementary table 6**. Oligonucleotides used in this work.

| **Oligonucleotide** | **Sequence (5’-3’)*** | **Purpose** | **Source** |
| --- | --- | --- | --- |
| nucSTB_CDC_S144R | ATTTCCACCGCGACCGAGCCACCTCGTTCGTCGCGGCACAGCAGGTCGACGGGTCCGATCGCGGTCATG | Oligo-mediated recombineering in the *nucS* gene to generate SNP S144R | This work |
| rpsL_K43R_TB | GCGGGCAACCTTCCGAAGCGCCGAGTTCGGCTTCCTCGGAGTGGTGGTGTACACGCGGGTGCATACACC | Oligo-mediated recombineering generating streptomycin resistance | (53) |
| Seq_1F_nucS_TB | ACGATGCCTCGCACCAGCGATCGTG | Amplification of *nucS* | This work |
| Seq_1R_nucS_TB | AGCGGTTTGCGGCGCCGAGCCATCG | Amplification of *nucS* | This work |
| Seq_2F_nucS_TB | TTCTCGGATGGCATGGTGACGTCAG | Sequencing *nucS* | This work |
| nucS ORBIT | tttgcggcgccgagccatcgcatcagtttaatcgcgcaactcagaacagccggtactcgccgctatccatGGTTTGTACCGTACACCACTGAGACCGCGGTGGTTGACCAGACAAACCctgggcgatgactagacgcacccgactcaccttagagcgcgcaacgacgttgttccttagagcgtgaccg | *nucS* ORBIT oligonucleotide to construct deletion *nucS* mutants | (37) |
| nucS CDS FW | GGTGAGTCGGGTGCGTCTAGTC | Verify the absence of *nucS* in the deletion *nucS* mutants | This work |
| nucS CDS RV | TCAGAACAGCCGGTACTCGCCGC | Verify the absence of *nucS* the deletion *nucS* mutants | This work |
| ORBIT nucS FW 5' | CAAGTGTCGAGTGGTGGCCTTCTCGG | Verify the 5’ junction in the deletion *nucS* mutants | This work |
| oriE Rv | CCTGGTATCTTTATAGTCCTGTCG | Verify the 5’ junction in the deletion *nucS* mutants | This work |
| new_nucS_EcoRI_F | AAAGAATTCGTGGGACTAGGAAAGCGCTG | Cloning *nucS* into pMV361 | This work |
| nucS_TB_HindIII_R | AAAAAAGCTTTCAGAACAGCCGGTACTCGC | Cloning *nucS* into pMV361 | This work |
| pMV_KO_Hsp60_Eco_R | AAAAGAATTCTCTAGATTTCTGGCGGGAAC | Removing promoter Hps60 and cloning *nucS* | This work |
| pMV_Hsp60_HindIII_F | AAAAAAGCTTATCGATGTCGACGTAGTTAA | Removing promoter Hps60 and cloning *nucS* | This work |
| cam gene FOR | TATCTGTTGTTTGTCCGGCCATCATGGCCGCGGTGATCAGTGATCGGCACGTAAGAGGTTCCA | *cam*^R^ gene for complementation of Δ*nucS*_TB_ mutants | This work |
| cam gene REV | GGTCTGCGATTCCGACTCGTCCAACATCAATACAACCTATGAAGATCCTTTGATCTTTTCTACGGGGT | *cam*^R^ gene for complementation of Δ*nucS*_TB_ mutants | This work |
| tsr gene FOR | TACCGCTCGCCGCAGCCGAACGACCGAGCGCAACGCGTGCGAATACTTCATATGCGGGGATCGACC | *tsr*^R^ gene for complementation of Δ*nucS*_TB_ mutants | This work |
| tsr gene REV | TGGTGCCCTTGGTGGTCGACTCTAGAGGATCCCCGGGTACACCGTTGACCTCGATATGTCAGT | *tsr*^R^ gene for complementation of Δ*nucS*_TB_ mutants | This work |
| RifRRDR-H37Rv Fw | GGTCGGCGAGCTGATCCAAAACCAG | Amplify the RRDR in the *rpoB* gene | This work |
| RifRRDR-H37Rv Rv | GCGGTACGGCGTTTCGATGAACCC | Amplify the RRDR in the *rpoB* gene | This work |

*Underlined sequences indicated overlapping sequences for the development of the Gibson assembly.

**Supplementary Figures.**

**
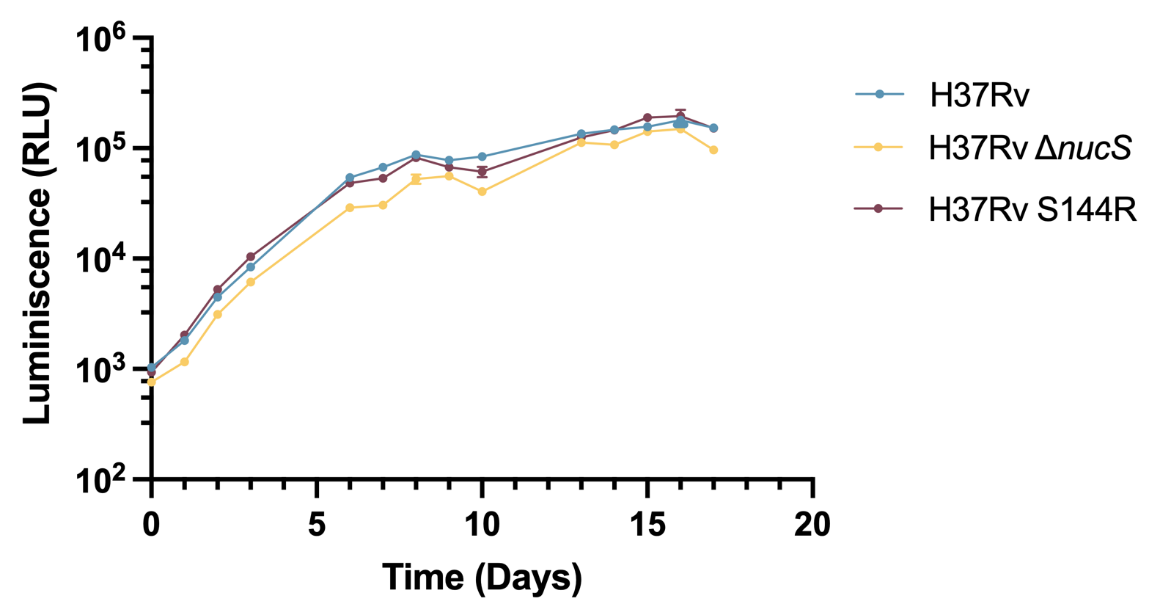
**

**Supplementary Figure 1.** Growth curves of *M. tuberculosis* H37Rv, H37Rv Δ*nucS* and H37Rv-S144R measured by nanoluciferase activity every 24 hours for a total of 18 days. Each point is the median value from four independent cultures. Error bars represent the SEM. Differences in maximum growth rates are not statistically significant (ANOVA followed by Tukey *post hoc* tests).

**
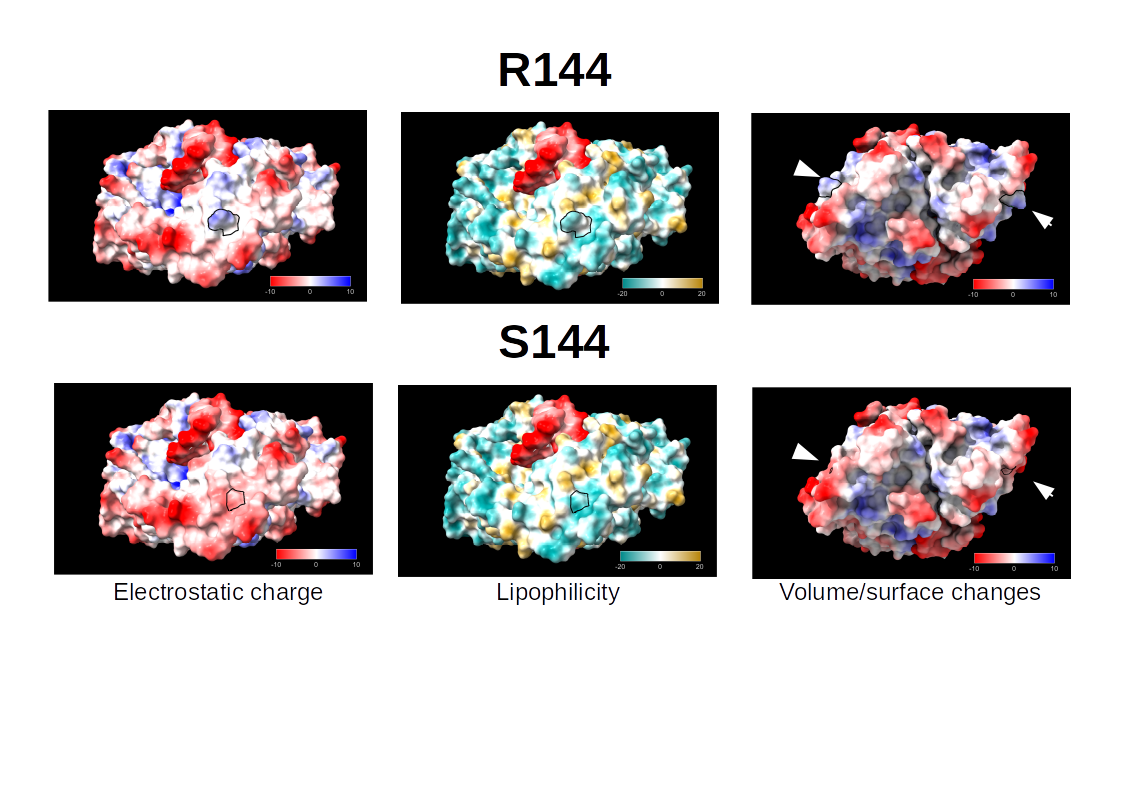
**

**Supplementary Figure 2:** Surface structural changes associated with arginine or serine in position 144 of NucS. The left and center columns show electrostatic charge and lipophilicity respectively, mapped on the protein-DNA complex surface, and oriented so that residue 144 is approximately centered (and delineated in black) to better highlight the changes they induce in their surrounding region. dsDNA stands out in red in the central panes. The right column shows electrostatic charge, but has been oriented to show both 144 residues -delineated in black and indicated by arrows- to highlight the volume changes in the protein surface. Substitution of R by S removes its positive charge and allows exposition of additional negative charge in its surrounding environment, induces lipophilicity changes, and causes a noticeable reduction in the exposed volume and surface area.

**
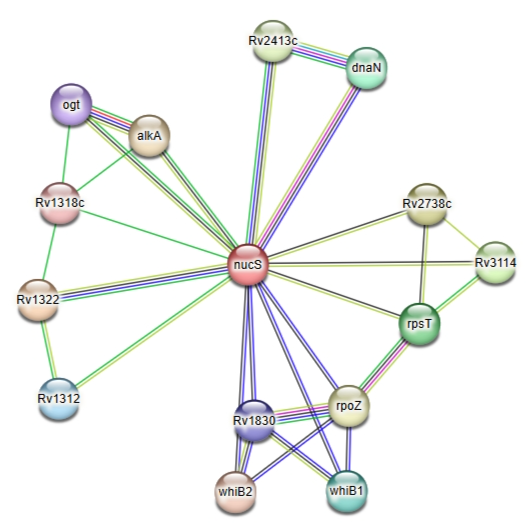
**

**
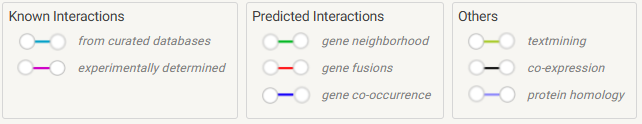
**

**Supplementary Figure 3**: Predicted *M. tuberculosis* H37Rv *nucS* interaction network computed using STRING v12.0 (<https://doi.org/10.5167/uzh-229577>). The network shows only predicted direct interactions, and was calculated using a high (score > 0.7) confidence level. Associations are meant to be specific and meaningful, i.e. proteins jointly contribute to a shared function; this does not necessarily mean they are physically binding to each other. The nodes are labelled with gene names and have been colored according to their grouping using k-means for four clusters. Edges are colored by interaction evidence.
